# Exploring the latest advancements in physical therapy techniques for treating cervical spondylosis patients: A narrative review

**DOI:** 10.17305/bb.2023.9049

**Published:** 2023-10-01

**Authors:** Quanzheng Chen, Zhenshan Wang, Shuna Zhang

**Affiliations:** 1 Department of Physical Education and Health, Guangxi Normal University, Guilin, China

**Keywords:** Cervical spondylosis, exercise therapy, rehabilitation, musculoskeletal manipulations, sports medicine

## Abstract

Cervical spondylosis is a widespread medical condition that significantly impacts patients’ quality of life. Treatment options include surgical and conservative approaches, with conservative treatment often being the preferred choice. Rehabilitation therapy is an essential component of conservative treatment, and advancements in technology have the way to the development of new physical therapy techniques. The effectiveness of treatment largely hinges on the patient’s ability to improve their dysfunction. This study aims to provide valuable insights into the use of new physical therapy techniques, such as sling exercises training, fascia manipulation, muscle energy technique, and proprioceptive neuromuscular facilitation that aid the rehabilitation of cervical spondylosis. By scrutinizing the current research status of these techniques, this study aims to present innovative ideas enhancing the rehabilitation process and outcomes for patients suffering from cervical spondylosis.

## Introduction

Cervical spondylosis is a degenerative disease that typically develops as a consequence of ligament and facet joint disorders. Its common symptoms include neck pain, numbness in the nerve root innervation area, nausea, vomiting, vertigo, and other related symptoms [1]. Cervical spondylosis commonly affects the C5-C6 and C6-C7 levels of the cervical spine. However, this condition can also result in high cervical spine lesions in some patients. The severity of the symptoms may vary based on the location and extent of the spinal damage [2]. Neck pain has been found to have a wide incidence rate ranging from 0.4% to 86.8% [3]. The prevalence of neck pain is higher among individuals who are at a higher risk of developing this condition. It is currently estimated that approximately 349 million people worldwide are affected by neck pain and related conditions [4]. Numerous studies have demonstrated that repetitive movements that exceed the normal range of motion in the joint can result in mechanical compression of the cervical spine, which may increase the risk of secondary injury [5]. Maintaining poor neck posture over an extended period can increase the burden on the cervical spine and accelerate the formation of chronic strain. Additionally, it can cause a forward head posture and lead to an increased incidence of neck pain [6, 7]. The treatment of cervical spondylosis typically involves surgical and conservative options, with conservative treatment being the primary approach. Recent research, both domestically and internationally, has proven physical therapy as an effective method for the prevention and treatment of cervical spondylosis, while also improving the quality of life for patients [8–10]. As rehabilitation medicine continues to advance, new physical therapy techniques have been developed and built upon traditional methods, offering unique advantages in the treatment of cervical spondylosis. This study explores the effectiveness of emerging physical therapy techniques in the rehabilitation of cervical spondylosis, including sling exercises training (SET), fascia manipulation (FM), muscle energy technique (MET), and proprioceptive neuromuscular facilitation (PNF). By examining the potential benefits of these techniques, this paper aims to offer novel insights and ideas for the diagnosis, treatment, and further research of cervical spondylosis.

## Anatomical basis of cervical spondylosis

**Figure 1.**
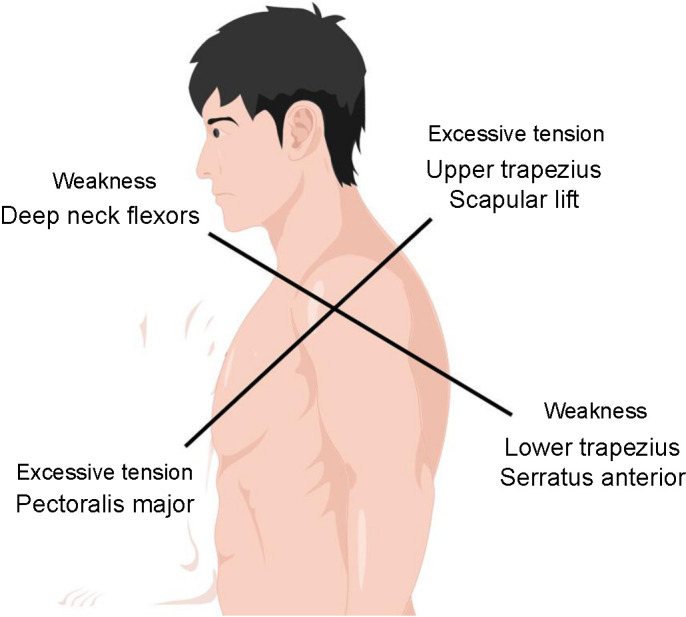
**Muscle strength imbalance in the upper crossed syndrome.** An example of strained and weakened muscles caused by superior cross syndrome: decreased muscle strength of deep cervical flexors, lower trapezius, and anterior serratus; excessive tension of upper trapezius, scapular lift, and pectoralis major; crossover of tight and weak muscle chains, and imbalance of muscle chains, resulting in abnormal posture [21].

The human cervical spine is the most flexible joint in the body, but it also has poor stability due to its structure. The vertebrae are interconnected by intervertebral discs (IVDs) and ligaments, except for the atlas (C1) and axis (C2) vertebrae and the sacral and coccygeal bones. The IVD is composed of the nucleus pulposus, annulus fibrosus, and cartilaginous vertebral endplate. As people age, IVDs become more susceptible to damage from external forces that compress and twist them. This can cause the annulus fibrosus to rupture, leading to cervical spondylosis. Normally, IVDs can withstand significant pressure, but age-related changes make them more vulnerable to damage [11, 12]. Some studies have found receptors similar to Golgi bodies in cervical discs [13]. There is some evidence to suggest that Ruffini bodies, which are mechanoreceptors found in the annulus fibrosus of IVDs, may play a role in causing dizziness in patients with cervical spondylosis [14]. Studies have shown that patients with cervical spondylosis often exhibit changes in their muscle structure and function. Paliwal et al. [15] found that these patients may experience reduced innervation of the relevant neck muscles and decreased nerve conduction velocity, which can ultimately lead to secondary muscle degeneration. Damage to the corresponding vertebral segments and adjacent facet muscles can lead to the development of neck pain [16, 17]. At the same time, a decrease in spinal stability can lead to degenerative changes in the spine, therefore, strengthening spinal stability is considered an effective treatment [18]. Cui et al. also discovered that patients with cervical spondylosis, poor posture, and forward head posture often have abnormal shoulder posture, resulting in upper cross syndrome (UCS) [19, 20]. Usually, UCS shows asymmetrical muscle strength of weak and strong muscles, decreased muscle strength of deep cervical flexors, lower trapezius, and anterior serratus; excessive tension of upper trapezius, scapular lift, and pectoralis major; crossover of tight and weak muscle chains, and imbalance of muscle chains, resulting in abnormal posture [21] (Figure 1)**.** If left untreated, these symptoms greatly impact a patient’s quality of life and worsen the progression of cervical spondylosis [22].

## Traditional physical therapy techniques

Traditional physical therapy techniques have been utilized for many years and have been effective in aiding patients in their recovery from various ailments. These techniques include strength training, skill training, heat therapy, acupuncture, tai chi, functional reconstruction, physical factor therapy, and many others. In the treatment of cervical spondylosis, traditional physical therapy has shown to be particularly effective in improving patient outcomes [23]. While traditional physical therapy techniques have been widely used for years to treat cervical spondylosis, they have some drawbacks. One important shortcoming is that the effects of traditional physical therapy may not be immediately noticeable, and patients may require ongoing treatment sessions to achieve relief of pain and other symptoms. This can make the treatment process quite lengthy [24]. In addition, traditional physical therapy can be quite costly, making it inaccessible to many people who do not have the financial means to undergo such treatment. Furthermore, traditional physical therapy may not focus on long-term prevention or resistance training, which can limit the long-term benefits for patients.

## Overview and application of the novel physical therapy techniques and technology

Novel rehabilitation physical therapy techniques integrate the latest technology to help enhance patient outcomes. For instance, suspension technology utilizes rope structure training to boost the connection between the patient and therapist [25], resulting in a more engaging and interactive rehabilitation experience. Advanced technology allows therapists to develop personalized treatment plans based on each patient’s unique needs and progress, leading to more targeted therapy that can accelerate the recovery process and improve overall outcomes. Additionally, patients tend to be more engaged due to the interactive nature of the therapy sessions, which can help them stay motivated and committed to achieving their recovery goals. Although some of these new rehabilitation techniques may come with a higher cost, in the long run, they can help speed up recovery time and decrease the need for additional treatment, making it more cost-effective for patients.

### Sling exercises training

#### Sling exercises training overview

SET is a unique physical therapy technique that was first introduced by Meier and further developed by Kirkesola. Originally, it was used to treat orthopedic conditions, but it has since been expanded to address rehabilitation training for sports injuries in humans [26]. The SET method maximizes the patient’s motor control by utilizing various tools and objects during the rehabilitation process. This allows the patient to move their body within a range that is free from pain or discomfort. The therapist provides assistance with both hands during the treatment to alleviate any pain or discomfort that the patient may experience [27]. Research has shown that SET is effective in improving muscle activity [28]. During upper limb exercise training assisted by SET, therapists conduct open and closed chain exercises on an unstable plane, while adjusting the patient’s body angle and gradually increasing the training difficulty. This activates the deep muscles, improves coordination between superficial and deep muscles, and uses postural reflexes to regulate muscle tension. The abnormal neuromuscular control mode of muscle groups is corrected, resulting in improved balance, sensation, and motor control abilities. Furthermore, this can aid in weight loss and core strengthening, ultimately improving core stability [25, 29]. The innovative weight loss technique utilized in SET can minimize the impact of gravity on the muscles and reduce the stimulation of pain receptors on the annulus fibrosus. This results in an increased range of joint movement, muscle relaxation, improved blood circulation, and enhanced elimination of inflammatory factors and metabolites, ultimately reducing pain. Additionally, the suspension belt provides additional stimulation to the joint capsule and ligament proprioceptors, enhancing their sensitivity and ensuring accurate sensory input, thereby improving neuromuscular control [30].

#### Sling exercises training application research

The majority of studies indicate that SET is effective in addressing muscle imbalances and improving core stability, particularly, in the neck region. In a randomized controlled trial involving women with neck pain, Park et al. [31] found that after four weeks of SET twice a week, there was a significant decrease in both cervical disability index and pain index, suggesting that SET can effectively alleviate neck pain and enhance quality of life in women. In a study conducted by Yan et al. on female patients with neck pain, it was discovered that four weeks of SET training could improve the condition of the neck muscle group and alleviate neck pain. This improvement may be attributed to better control of the neck nerves [32]. Wang et al. [33] discovered that SET training has the ability to activate deep neck muscles and enhance core stability, which can strengthen the weak chain that contributes to UCS (muscle weakness), weaken the strong chain, restore the balance of muscle tension, improve abnormal posture, and enhance the cervical spine’s anti-fatigue ability. Several studies have indicated that SET exercises are effective in improving the cervical range of motion, although some angles may not be as effective as traditional stretching exercises [34]. Research has demonstrated that SET is more effective in relieving neck pain compared to other treatments, however, it may not be as effective in improving the cervical range of motion as other techniques. While SET has been extensively used to treat patients with lower back pain and stroke, there is still a dearth of high-quality studies on its effectiveness in treating cervical spondylosis. As the joint with the largest range of motion in the human body, it is essential to improve the stability of the cervical spine and reduce pain while maintaining its flexibility [18]. Studies have suggested that SET is effective in enhancing core stability, which can effectively treat cervical spondylosis. Therefore, it is important to further explore the feasibility and mechanisms of SET as a treatment option for cervical spondylosis. Currently, there is a lack of sufficient research on the relationship between SET and neuromuscular control mechanisms. In future studies, it may be beneficial to use precision instruments such as electromyography to investigate the neural plasticity of SET.

### Fascia manipulation

#### Fascia manipulation overview

Fascia is a type of connective tissue that includes tendons, ligaments, and joint capsules, among other structures. Cervical spondylosis is often accompanied by symptoms, such as dizziness, neck pain, and eye pain. According to the fascia theory, the proximal posterior fascia chain is made up of the deep fascia of the neck and the fascia sheath of the eye. Therefore, when the fascia is damaged, it can cause discomfort in the corresponding areas [35, 36]. Additionally, neck pain and other issues may result from irritation of joints, ligaments, fascia, and other tissues. Studies have found that the myofascial membrane is capable of transmitting force to other muscle groups. From a physiological perspective, the sarcolemma efficiently transfers force through transmembrane substances to the extracellular matrix, which ultimately terminates in the tendon [37]. The conduction of fascia is not solely dependent on itself, but it can also be influenced by external forces. Manipulative treatments can apply internal forces that may alter its tension [38]. FM enhances muscle elasticity by improving the flexibility of fascia. It can also enhance Ca^2+^ reactivation while reducing muscle pain caused by delayed onset muscle soreness that occurs 24 h after exercise [39]. The therapist uses their elbow or knuckle to manipulate the fascia in a specific area, restoring its ability to glide smoothly [40].

#### Fascia manipulation application research

FM has recently acquired popularity in the treatment of sports injuries and has shown promising results. Among the various types of cervical spondylosis, radicular cervical spondylosis is a common occurrence, accounting for about 60%–70%. Its primary symptoms include upper limb weakness and limb numbness. In the case of limb numbness, the pressure on the adjacent tissues in the innervated area of brachial plexus is considered a crucial factor. The brachial plexus is enveloped by a sheath, which plays a vital role in determining the speed and recovery of nerve conduction. In a study conducted by Turazza et al., it was shown that FM can impact the sheath tone of the brachial plexus, improve patients’ proprioceptive ability, and thereby promote nerve restoration, leading to an improvement in the symptoms of upper limb numbness [41]. Dos Santos Amorim et al. employed near-infrared spectroscopy to assess the level of oxygenated hemoglobin in the trapezius muscle tissue of the subjects. The results showed that FM could enhance the blood circulation in the local treated area, leading to a significant increase in the oxygenated value of the trapezius muscle in the treated patients. These findings suggest that FM has remarkable benefits in this area [42]. Rodriguez-Huguet et al. discovered that patients with neck pain experienced significant improvements in pressure pain threshold and digital pain score after undergoing two weeks of fascia therapy. Furthermore, the efficacy of this treatment was found to be superior to that of conventional standard physical therapy [43]. Fascia therapy is a relatively new field, and there is limited research on its effectiveness for treating cervical spondylosis. The physiological and biochemical mechanisms involved in the treatment of cervical spondylosis with fascia therapy are still poorly understood. Further research can be conducted to investigate the physiological and biochemical processes involved in the treatment, and to better understand the mechanisms through which the muscle pain can be alleviated.

### Muscle energy technique

#### Muscle energy technique overview

MET is an energy-based therapy that aims to adjust abnormal muscle tone by training specific muscles, enhance muscle strength and stability in corresponding areas, improve the musculoskeletal system’s function, and ultimately improve patients’ quality of life [44]. Common techniques used in MET include centrifugal contraction, reciprocal inhibition, contraction–relaxation, contraction–relaxation–contraction, and others. MET not only improves muscle strength and elasticity but also enhances core stability and motor control [45]. During MET, patients follow the therapist’s instructions and engage in antagonist exercises with the therapist to relax spasmodic muscles, strengthen weakened muscles, adjust the tension balance, and reduce pain [46]. Numerous studies have indicated that MET can effectively promote cell synthesis, blood flow, lymphatic circulation, accelerate substance metabolism, improve tissue excitability, and prevent muscle atrophy [47]. MET stimulates the Golgi tendon organ through active muscle isometric contraction, and the Golgi tendon organ that converts the signal into impulses that are transmitted to the α motor neuron controlling the muscle. This process inhibits the muscle spindle receptor and relaxes the muscle through negative feedback regulation [48]. Isometric contraction of muscles leads to sliding of coarse and fine muscle filaments against each other in the sarcomere, generating heat and increasing the ductility and elasticity of surrounding tissues. This leads to an increase in muscle malleability, reduction in muscle tension, and relief from body pain.

#### Muscle energy technique application research

In a study conducted by Sbardella et al., it was observed that patients suffering from acute and chronic neck pain showed significant improvement in cervical range of motion after undergoing MET training. The improvement was even greater when combined with traditional treatment methods [49]. This suggests that MET can be an effective technique in correcting abnormal posture and improving body function, particularly in patients with neck pain. In a study by Joshi et al. on neck pain, it was found that after three weeks of MET training, patients showed significant improvements in cervical disability scores and cranial vertebral angle. However, because there was no separate group for MET training in literature, it is difficult to determine the effectiveness of this treatment compared to other treatments [50]. Despite the positive results of MET in improving cervical range of motion and reducing neck pain, its application in treating cervical spondylosis is still limited, with little research available. Currently, there is no standardized protocol for the application of MET in the treatment of cervical spondylosis, including the direction and angle of application, resistance, total repetitions, and extent of application. Although the improvement of muscle function by MET training may be related to the nervous conduction system, further research is needed to clarify the specific conduction system involved and its effects and future studies should focus on the neurological effects of MET. Moreover, while MET is often used in combination with other physical therapy methods for treating cervical spondylosis, there is insufficient evidence to determine its effectiveness compared to other approaches. Therefore, the next step in further research should be the comparison between the short-term and long-term efficacy of MET with other physical therapy methods.

### Proprioceptive neuromuscular facilitation

#### Proprioceptive neuromuscular facilitation overview

PNF is a treatment technique that was developed in the 1950s by Tedla and Sangadala [51]. PNF is a rehabilitation technique that involves stimulating proprioceptors in a spiral diagonal pattern to enhance the neuromuscular response and improve muscle contraction ability. It also involves adjusting abnormal sensory nerve excitability to normalize muscle tension and movement patterns [52, 53]. PNF can be applied in various areas, such as the pelvis, upper limbs, and lower limbs, to improve patients’ motor function and aid in their rehabilitation. The basic techniques of PNF include rhythmic initiation, contract–relaxation, isotonic combination, dynamic reversal, and many other techniques. These techniques can help stimulate weak muscles and promote muscle contraction in various ways [54]. Resistance training in PNF can help increase the strength of respiratory muscles, activate proprioceptors in the respiratory muscles, induce reflex breathing movements, and improve blood circulation [55]. PNF can improve the range of motion of patients with soft tissue injuries by using a multi-level and multi-directional upper limb movement mode. Additionally, it can effectively enhance muscle strength, balance ability, coordination ability, and promote nerve recovery [56, 57]. PNF works on the principle of increasing stimulus intensity leading to an increase in the body’s response intensity. Even after the stimulus stops, the response continues to exist due to the effects of continuous static contraction. This is likely why PNF exercises have been shown to improve joint motion, establish new neural pathways, promote neural plasticity, enhance limb function, and facilitate limb rehabilitation, especially in shoulder joint PNF exercises according to existing research.

**Table 1:** Comparison of conventional and novel treatments for cervical spondylosis. **Comparative analysis of traditional and new therapies in terms of safety, cost-effectiveness, treatment, application range, and therapeutic effect**

**Comparison**	**Safety**	**Cost-effectiveness**	**Treatment**	**Application range**	**Therapeutic effect**
Traditional treatment	Relatively safe	Relatively low	Relatively long	Wide	Effective
PNF	Safe	Relatively high	Relatively short	Wide	Effective
SET	Safe	–	Relativel y short	Limited	Effective
FM	Safe	–	Relatively short	Limited	Effective
MET	Safe	–	Relatively short	Limited	Effective

“–” represents unclear data; PNF: Proprioceptive neuromuscular facilitation; SET: Sling exercises training; FM: Fascia manipulation; MET: Muscle energy technique.

#### Proprioceptive neuromuscular facilitation application research

As a treatment technique commonly used in the field of musculoskeletal rehabilitation, PNF was initially developed for use in neurological diseases, such as stroke, multiple sclerosis, and poliomyelitis, but it has recently gained attention for its unique effectiveness in treating cervical spondylosis [58, 59]. A study conducted by Ashfaq et al. on patients with neck pain randomly assigned subjects to three groups: PNF+vertebral passive movement (PVM), PVM+conventional physical therapy (RPT), and RPT alone. After four weeks of intervention, the PNF+RPT group showed less improvement in the patients’ daily life quality compared to the PVM+RPT group [60]. In this study, since no separate PNF treatment group was established, it was not possible to determine the effectiveness of PNF compared to routine physical therapy. However, Yang and Qiao [61] found that when PNF was combined with massage, release, and routine physical therapy, it effectively alleviated neck and shoulder muscle imbalance and improved poor postures, such as rounded shoulders and hunchbacks in college students. This improvement may be due to the unique spiral diagonal crossover pattern of PNF. By challenging the core through upper limb movements, distal activity training emphasizes proximal stability, weakens tense muscles, strengthens weak muscles, and restores muscle symmetry [62], thereby improving poor postures, such as rounded shoulders and hunchback. The duration of treatment was not specified in the study, so it is unclear whether the effects of PNF were short term or long term. In summary, future research could focus on comparing the effectiveness of PNF with other treatments, as well as investigating both short-term and long-term outcomes.

## Discussion

PNF is a physical therapy technique that aims to improve neuromuscular function, flexibility, and strength [63]. Sling exercise training uses a suspension system to support the body and help practice, which can enhance core strength improve posture, balance, and reduce pain [25]. Fascial manipulation targets connective tissue to improve range of motion and reduce pain [64]. MET uses isometric contractions to lengthen tight muscles and improve joint mobility, enhancing the body’s strength and flexibility through muscle contraction [65]. Compared to conventional physical therapy, SET, FM, MET, and PNF appear to have several advantages in terms of safety, efficacy, and cost-effectiveness. However, SET, FM, and MET may have a slightly limited treatment scope compared to conventional treatment and PNF. For instance, while conventional treatment and PNF cover both musculoskeletal and neurological diseases, SET, FM, and MET may primarily target musculoskeletal diseases or some specific conditions [34, 49, 66–73], as shown in Table 1. Although the new treatment options represent new hope for patients with cervical spondylosis, their potential advantages and shortcomings need to be carefully considered. PNF, fascial manipulation, and MET require skilled medical personnel, and improper operation may lead to muscle strains. Suspension training requires special suspension devices that may not be readily available and may be less flexible. There are also few relevant studies with small number of subjects, and the evaluation indexes are mostly scales, which are not measured with more sophisticated instruments. Most of the treatment protocols are combined treatments, and it is unclear which treatments are more effective for which types of cervical spondylosis. Therefore, more high-quality randomized controlled trials are needed to study these treatments in the next step. Although the causes and mechanisms underlying the development of cervical spondylosis are not yet clear, it is well known that the painful distress caused by cervical spondylosis can be effectively resolved through a combination of various physical treatments that regulate the balance of muscle tone, strengthen core stability, and correct abnormal biological force lines. It is likely that new physical therapy technologies for the treatment of whiplash will be developed and refined in the future, such as virtual reality (VR) and wearable devices. In recent years, VR technology has been used in the field of pain management [74]. VR can be used to assess cervical spine range of motion and motion capacity by collecting information on angular velocity and displacement during movement of cervical spine patients [75]. Interactive modes using games or real-time feedback can reduce pain, improve joint range of motion and facilitate guidance for further rehabilitation of patients [76]. This may be related to the fact that VR effectively improves the coordination of deep and superficial neck muscle groups [77]. VR technology has a remote control function, which can to some extent overcome the space limitations of the new physical therapy technologies, facilitate the rehabilitation of patients at home, and reduce time costs. This demonstrates the promising potential of combining VR with new physical therapy technologies for the treatment of cervical spondylosis. VR can improve patient compliance and treatment effectiveness through an immersive experience. Additionally, VR provides information monitoring during treatment, allowing the real-time feedback and adjustment of treatment strategies for more precise and personalized treatment. Furthermore, it may reduce the burden of medical care, as VR technology applications may reduce the need for therapist assistance or specialized medical equipment.

## Conclusion

Overall, the combination of VR technology and other novel technologies has the potential to improve the field of pain management. However, further research is needed to explore the feasibility of these options and to compare their efficacy with traditional techniques. The development of technology has facilitated the faster dissemination of various technical ideas, and the integration of these ideas has given rise to various novel technologies. However, many of the mechanisms underlying the effects of these new technologies on the treatment of cervical spondylosis are still unknown. Therefore, it is necessary to focus on the study of their theoretical mechanisms and their effective application in the clinic to improve the rehabilitation treatment of cervical spondylosis.
